# Enteric fever in an HIV/AIDS patient: Atypical manifestations

**Published:** 2012-09

**Authors:** M Chatterjee, B Chakraborty, SS Chatterjee, M Bose, K Mukherjee, A Basu, S Das, M Banerjee, U Ghosh

**Affiliations:** Department of Microbiology, NRS Medical college, India

## Abstract

Bloodstream infections with *Salmonella typhi*, is uncommon in human immunodeficiency virus (HIV)-infected persons. The symptoms in such patients are often non-specific and have a rather insidious onset and progression. We report a patient with sepsis and lower limb gangrene due to *Salmonella typhi* infection in an HIV-infected patient.

## CASE REPORT

A 38-year old man presented to the medicine outpatient department of Nilratan Sircar Medical College and Hospital, Kolkata with irregular lowgrade fever for three weeks, progressive blackening and ulceration of fingers and toes for 15 days and altered sensorium for last 4 days. Patient was a taxi driver, married but with multiple sexual partners, and a chronic alcoholic (12 years) and smoker as well (20 years, 8-10/day).

**Fig. 1:**
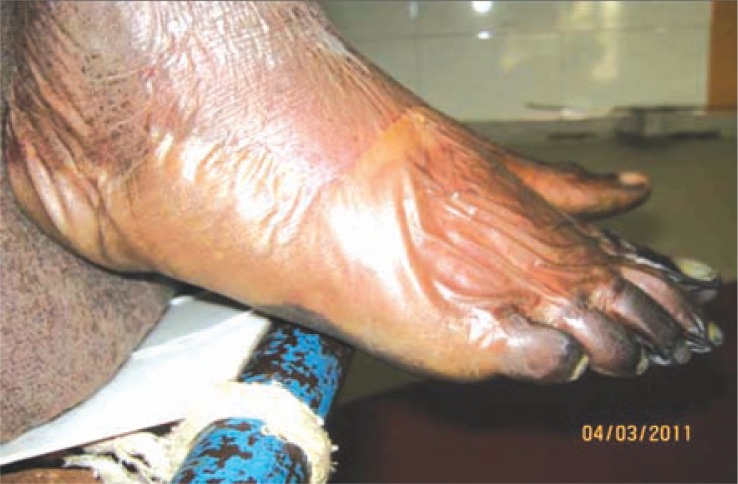
Right foot showing cellulitis, blackish discolouration of the foot, and collection of pus on the dorsum and medial side of the foot.

**Fig. 2:**
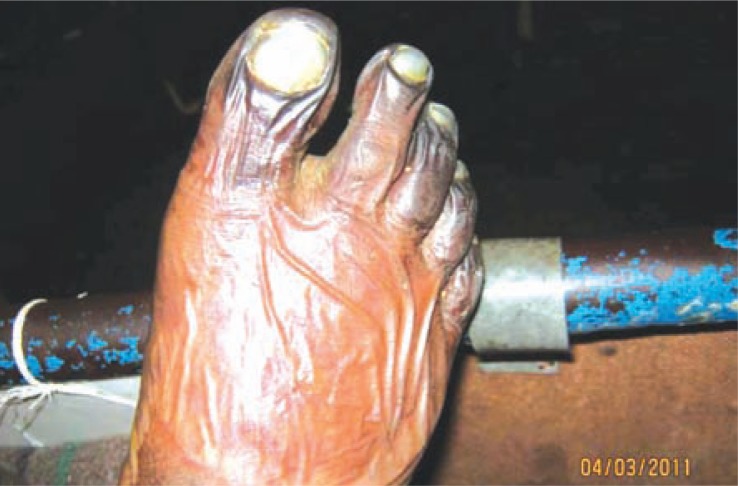
Gangrenous changes in the toes of the right foot.

 Clinical examination revealed pallor, drowsiness (Glasgow coma score - E4V4M4), pulse of 120 /min (regular), blood pressure of 116/78 mm Hg, elevated temperature (103.4°F), but normal respiratory rate. Peripheral pulses were absent in the right popliteal and dorsalis pedis. There were no localizing neurological signs, no neck rigidity and plantars were flexor. Cardiovascular system and chest showed no abnormality. Tongue appeared red, however papillae were normal and without any ulcers. Liver and spleen were not palpable. The right lower limb revealed cellulitis, blackish discolouration of the foot, and collection of pus on the dorsum and medial side of the foot (Fig. 1). Gangrenous changes were distinct especially on the 3^rd^, 4^th^, and 5^th^ toes (Fig. 2); while the rest of the foot was oedematous. The left middle and index fingers were also gangrenous (Fig. 3A). Loss of skin over the distal phalanges of the right index (Fig. 3B) and left middle finger of the hands were also noticed. Patient gave a past history of genital lesion (suggestive of chronic balanoposthitis) which occurred 15 years ago, which healed with medication. Presently the genitalia are healthy.

Peripheral blood revealed - Haemoglobin 9.1 gm/ dl, WBC count of 9000/cu mm, with a differential of neutrophil 80%, lymphocyte 12%, eosinophil 1%, and monocyte 7%; Mean Corpuscular Volume was 96.8, Mean Corpuscular Haemoglobin Concentration 33.3 and platelet count was 80000/cu mm. Coagulation profile, fasting blood sugar, creatinine, urea, and uric acid were within normal limits. Liver function tests showed normal values except altered A:G ratio (0.86). HBsAg and anti-HCV were negative. Routine examination of urine and stool revealed no abnormality.

Cerebro-Spinal Fluid study showed normal opening pressure, normal cell count (10/cumm, mostly lymphocytes), normal sugar (106 mg/dl), protein (34 mg/dl), and adenosine deaminase (2.4 U/dl) with negative results for fungal element or acid fast bacilli. The patient was empirically started on ceftriaxone (2 gm IV twice daily), metronidazole (400 mg IV thrice daily) and enoxaparin (40 mg twice daily). Fever decreased to low grade after 4 days of treatment but mental status showed no improvement.

He was found reactive for HIV-1 as per National AIDS Control Organization guidelines. Blood was sent for culture on the 3^rd^ day post admission. Even though the patient was already on antibiotics, on the 2^nd^ day of incubation, culture showed growth of *Salmonella typhi*. It was sensitive to chloramphenicol, ofloxacin, ceftriaxone, ciprofloxacin, co-amoxyclav and imipenem by standard disc diffusion testing.

**Fig. 3A:**
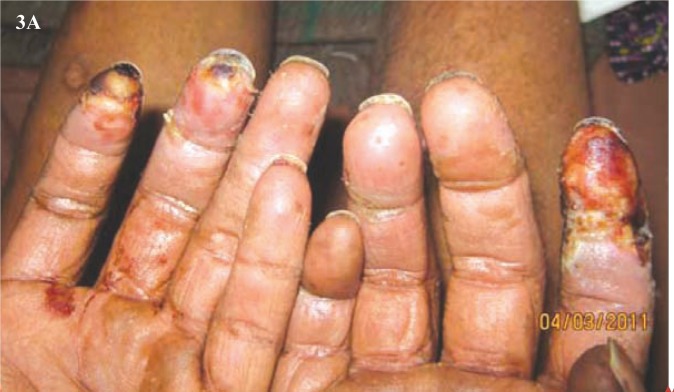
Gangrenous changes in the tips of the left middle and index fingers.

**Fig. 3B:**
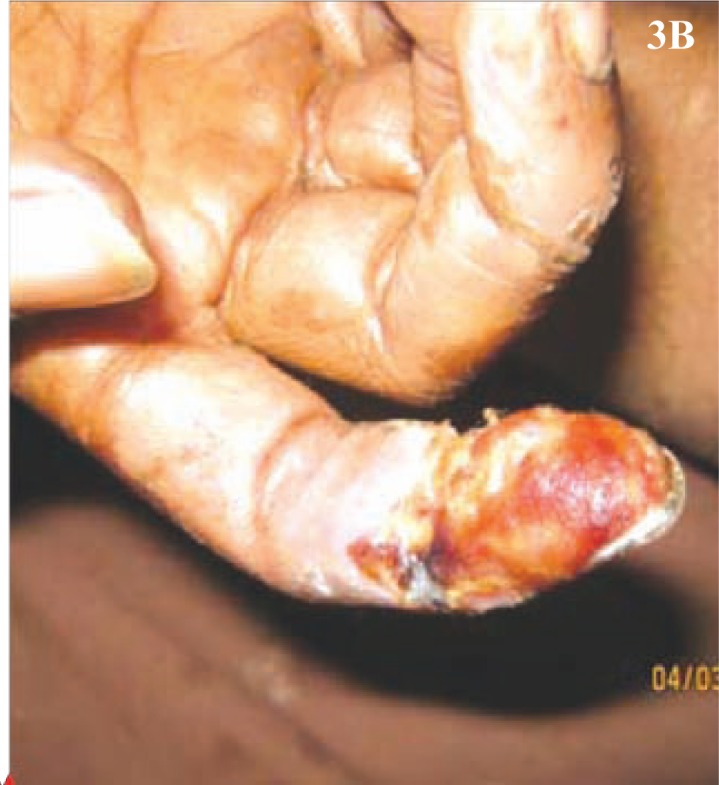
Loss of skin over right index finger.

Pus collected from the abscess on the foot and gangrenous areas revealed plenty of pus cells and gram positive cocci in clusters on gram stain; culture yielded growth of *Staphylococcus aureus* sensitive to cefotaxime, cloxacillin, ceftriaxone, vancomycin, linezolid, clindamycin, clarithromycin, amikacin, gentamicin, and netilmicin. There was no inducible resistance to clindamycin or clarithromycin. There were no acid fast bacilli and anaerobic culture showed no growth. KOH preparation of pus revealed no fungal element, and fungal culture was sterile after 6 weeks of incubation. Sputum was negative for acid fast bacilli.

Serological test for cryptococcal antigen and VDRL were negative. His CD 4 count was 160/µl. Straight X-ray chest and abdominal sonography were normal and the mantoux test was non reactive. Ultrasonography with Doppler study of both lower limb arteries expressed a biphasic wave pattern in popliteal and tibial vessels and no flow in dorsalis pedis along with moderate atherosclerotic changes-all features more prominent on the right side. Anti-phospholipid antibodies were negative.

Injection ceftriaxone was continued, oral cotrimoxazole and of meropenem (1 gm IV thrice daily) were added and continued for another ten days. The patient improved, his mental status became normal, and repeat blood culture was negative after 12 days. The abscess on his leg dried but the gangrenous zone persisted, and an amputation was planned. Stool and urine culture (to detect carrier stage for *Salmonella typhi*) were negative. After this he was put on triple drugs, (Zidovudine, Lamivudine and Nevirapine).

## DISCUSSION

*Salmonella* bacteriaemia is a known opportunistic infection occurring in HIV infected patients. Despite the high prevalence of *Salmonella typhi* in developing countries (India), an association between HIV and *S. typhi* has not been well documented and reports are infrequent. In most reports, infections are due to non-*typhi* salmonellae mainly *Salmonella typhimurium* and *enteritidis* (1–5).

*Salmonella typhi* infection in HIV/AIDS patients may cause life threatening complications (6) like septic shock, meningitis, as also local abscess formation. Though meningococcemia is the most common infection resulting in peripheral gangrene, other infectious agents like *S. typhi* have been implicated (7, 9). Proposed pathogenic mechanisms for development of peripheral gangrene in systemic infections include the Schwartzman reaction, bacterial endotoxin release, platelet sludging due to vascular collapse and Disseminated intravascular coagulation (DIC) (8). *Salmonella typhi* infection has also been reported to cause myocarditis and large vessel thrombosis, involving the femoral artery (10). Typhoid fever is relatively common in India but occurrence of peripheral gangrene is exceedingly rare (7). In our patient, endotoxemia, resultant hypotension and platelet sludging may have been responsible for the symmetrical gangrene in the absence of abnormal DIC studies or any evidence for embolization.

The patient presented with prolonged low-grade irregular fever which is an atypical presentation of Enteric fever. Further isolation of *S. typhi* after two days of empirical therapy with ceftriaxone to which the bacterium was in-vitro sensitive is intriguing. Though the bacterium persists in bone marrow for a period of 5 days or more despite correct antimicrobial therapy. Its persistence in blood is not reported in literature. It is likely that the patient's immunosuppressed status contributed to this finding.

The purpose of reporting this case is the atypical presentation of enteric fever and gangrene due to *Salmonella typhi* and superinfection with *staphylococcus aureus* in a first time detected HIV reactive patient. This report is to alert clinicians about this rare complication of a common curable disease in a HIV/AIDS patient, so that appropriate preventive measures can be provided.

## References

[1] Arthur G, Nduba VN, Kariuki SM, Kimari J, Bhatt SM, Gilks CF (2001). Trends in bloodstream infections among human immunodeficiency virus-infected adults admitted to a hospital in Nairobi, Kenya, during the last decade. Clin Infect Dis.

[2] Mtove G, Amos B, von Seidlein L, Hendriksen I, Mwambuli A, Kimera J (2010). Invasive salmonellosis among children admitted to a rural Tanzanian hospital and a comparison with previous studies. PLoS One.

[3] Fernandez Guerrero ML, Ramose JM, Nunez A, Nunez A, de Gorgolas M (1997). Focal infections due to non typhi Salmonella in patients with AIDS: Report of 10 cases and review. Clin Infect Dis.

[4] Brent AJ, Oundo JO, Mwangi I, Ochola L, Lowe B, Berkley JA (2006). Salmonella bacteremia in Kenyan Children. Pediatr Infect Dis J.

[5] Gordon MA (2008). Salmonella infections in immunocomromised adults. J Infect.

[6] Manfredi R, Chiodo F (1999). Salmonella typhi disease in HIVinfected patients: case reports and literature review. Infez Med.

[7] Passi GR, Singsit J, Singh M (1997). A 12-year-old boy with fever and blue ears. Indian Pediatr.

[8] Molos MA, Hall JC (1985). Symmetrical peripheral gangrene and disseminated intravascular coagulation. Arch Dermatol.

[9] Adeyokunnu AA (1982). Bilateral gangrene of the feet associated with *Salmonella* infection in children with sickle cell anemia. Ann Trop Pediatr.

[10] Okoko BJ, Ota MO, Arowolo JO, Whittle HC (2001). Peripheral gangrene complicating Salmonella typhi septicaemia in a Gambian infant. J Trop Pediatr.

